# Supplementary material for: Enhanced subglacial discharge from Antarctica during meltwater pulse 1A
Source: Nat Commun. 2023 Nov 13;14:7327. doi: 10.1038/s41467-023-42974-0 (PMC10643554; doi:10.1038/s41467-023-42974-0)
Supplement: Supplementary file 1 — Supplementary Information [file 41467_2023_42974_MOESM1_ESM.pdf]

Supporting Information for

Enhanced subglacial discharge from Antarctica during meltwater pulse 1A

Tao Li*, Laura F. Robinson, Graeme A. MacGilchrist, Tianyu Chen, Joseph A. Stewart, Andrea Burke, Maoyu Wang, Gaojun Li, Jun Chen, James W.B. Rae
*correspondence to: taoli@nigpas.ac.cn

This PDF file includes:

Supplementary Figure 1 to 8

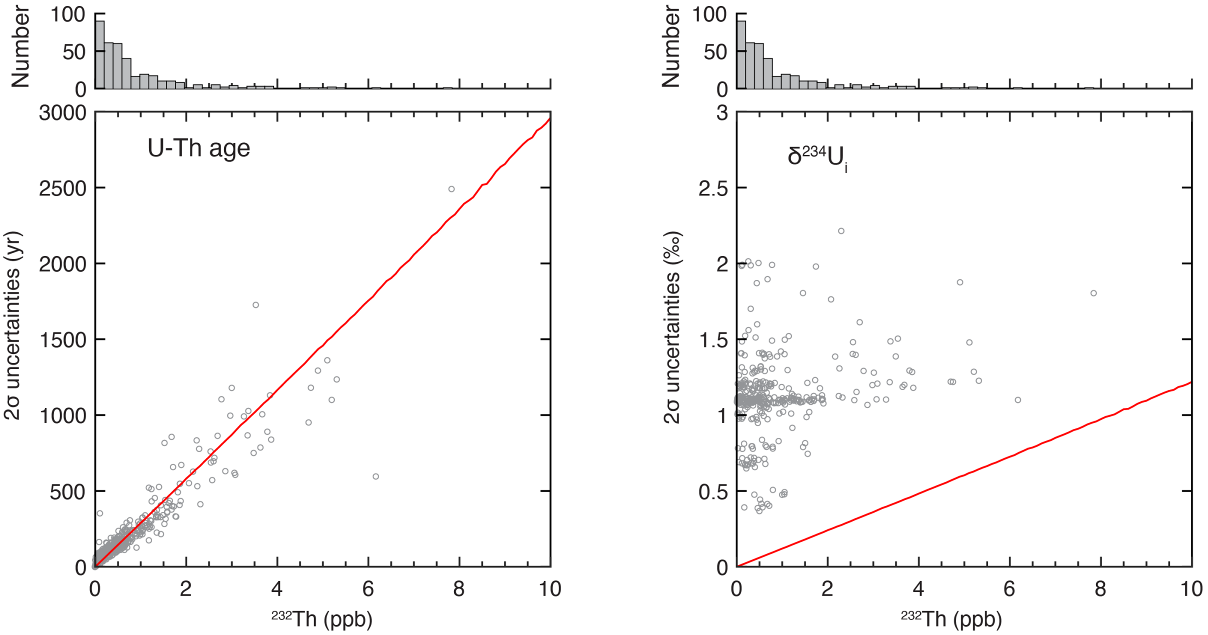

Supplementary Figure 1 | Influence of initial ^{230}Th correction on final age and $\delta^{234}U_i$ uncertainties. Upper panels: histogram of deep-sea coral ^{232}Th contents. Lower panels: grey circles represent measured data for deep-sea coral samples and red lines indicate modeled uncertainties for both age and $\delta^{234}U_i$ for a modern sample (age = 0 year, $\delta^{234}U$ = 146.8‰) and a 20000-year-old sample ($\delta^{234}U_i$ = 146.8‰) with varying ^{232}Th contents. The modeled uncertainties were calculated by considering a relatively large uncertainty in the modern-day $^{230}Th/^{232}Th$ atomic ratio ($2\pm2 \times 10^{-4}$, 2σ)[1] and were propagated with a Monte Carlo method when solving the age equation[2-4].

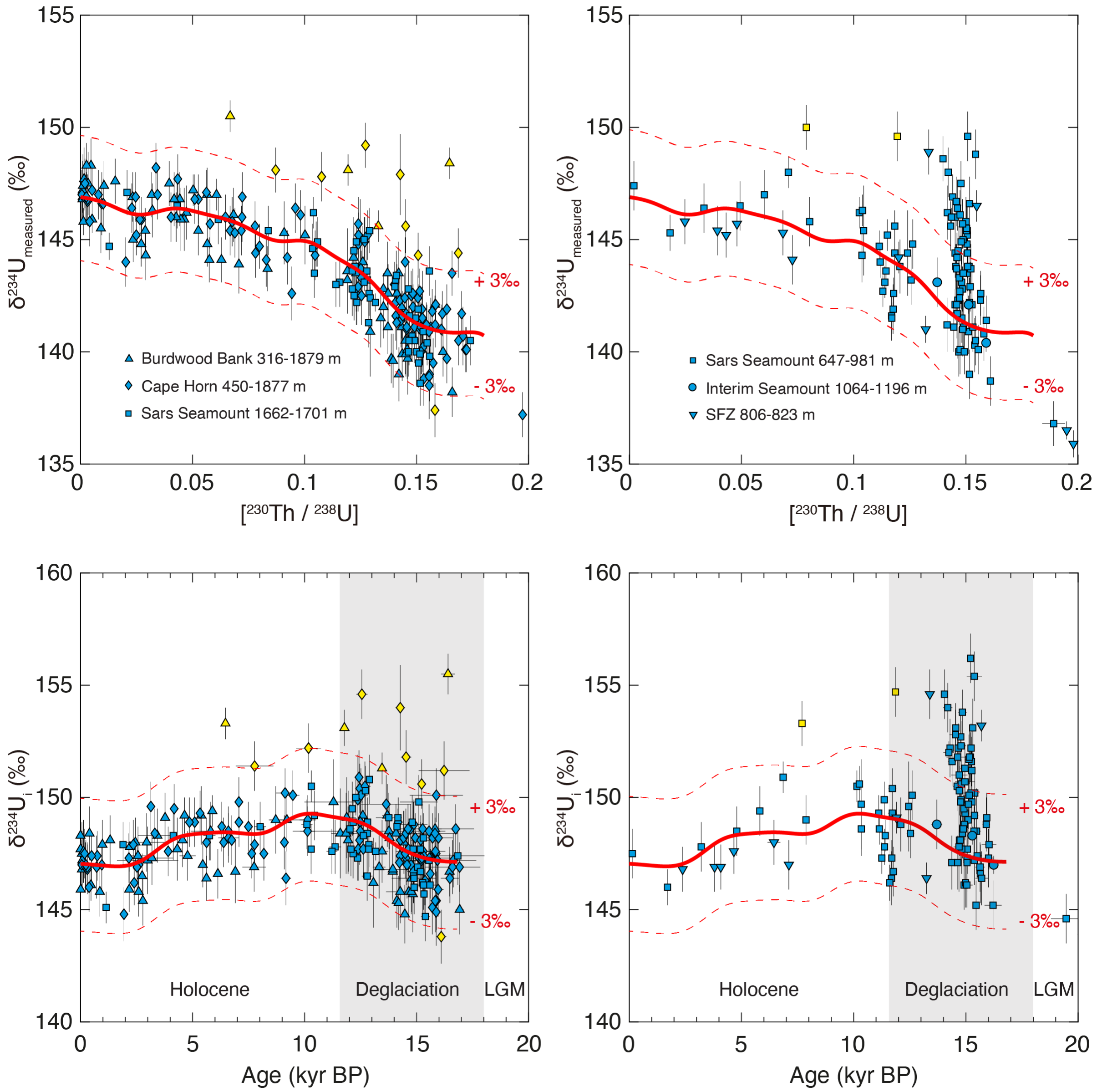

Supplementary Figure 2 | Compiled U-series data of the Drake Passage deep-sea coral. Error bars represent 2σ uncertainties. Red lines enveloped by dashed lines (± 3‰) denote the smoothed seawater $\delta^{234}U$ record at the Drake Passage and the upper and lower limits of seawater $\delta^{234}U$. Yellow symbols represent deep-sea coral samples characterized by abnormal $\delta^{234}U_i$ values (n = 14) which were omitted in our final dataset (Methods).

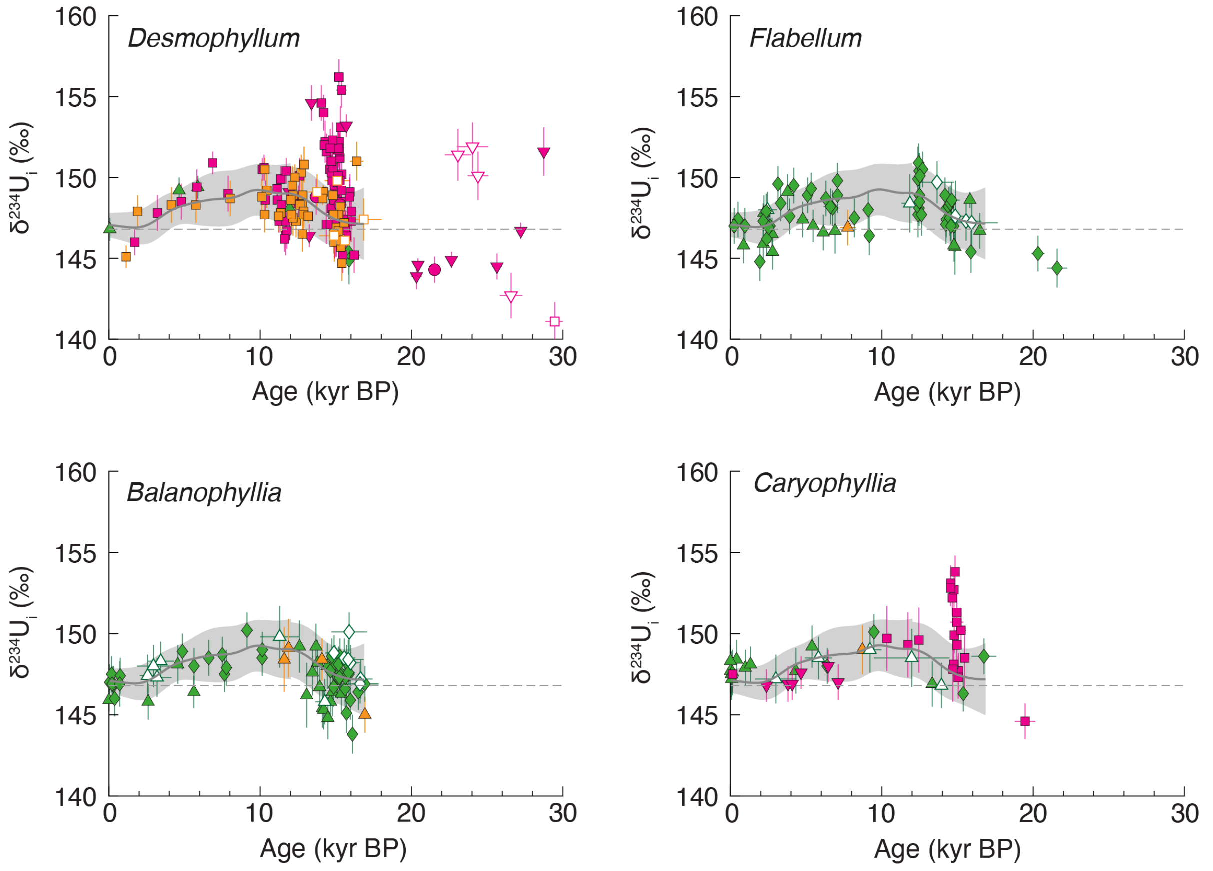

Supplementary Figure 3 | The $\delta^{234}U_i$ records for different deep-sea coral genera. Symbols with 2σ uncertainties are the same as in Fig. 2. Open symbols represent samples with a ^{232}Th concentration higher than 2 ppb. Black lines enveloped by grey shading (± 2σ uncertainties) denote the smoothed $\delta^{234}U$ record (excluding samples from Sars Seamount 647-981 m, Interim Seamount 1064-1196 m, and Shackleton Fracture Zone 806-823 m) with a 500-yr Gaussian filter. Horizontal dashed lines mark the $\delta^{234}U$ value of modern seawater[5].

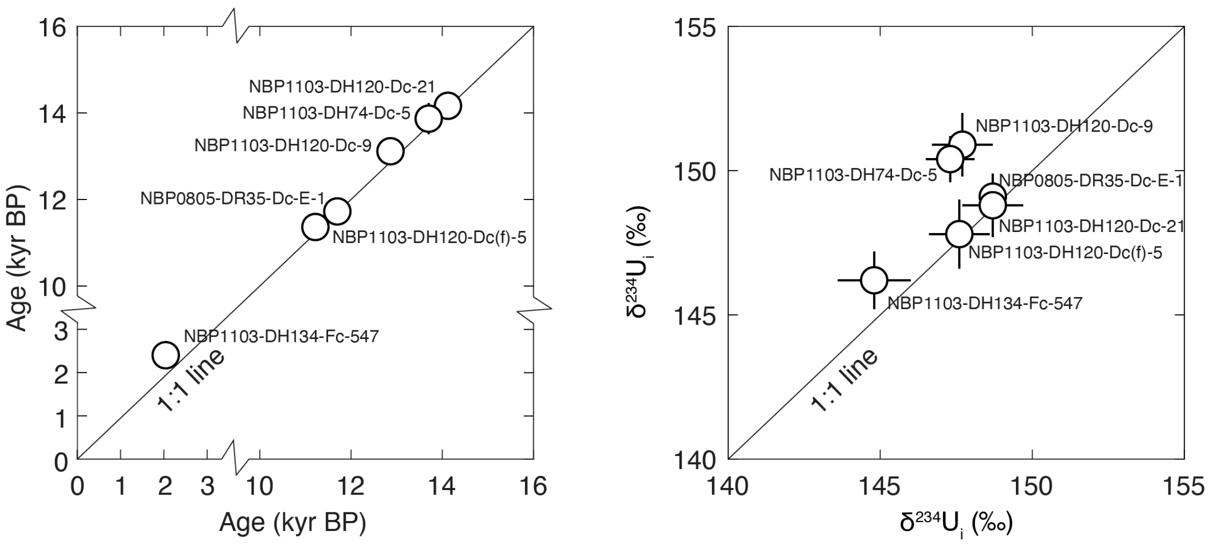

Supplementary Figure 4 | Replicate measurements of six deep-sea coral samples showing consistent age results but $\delta^{234}U_i$ differences of up to ~3 ‰. Error bars represent 2σ uncertainties.

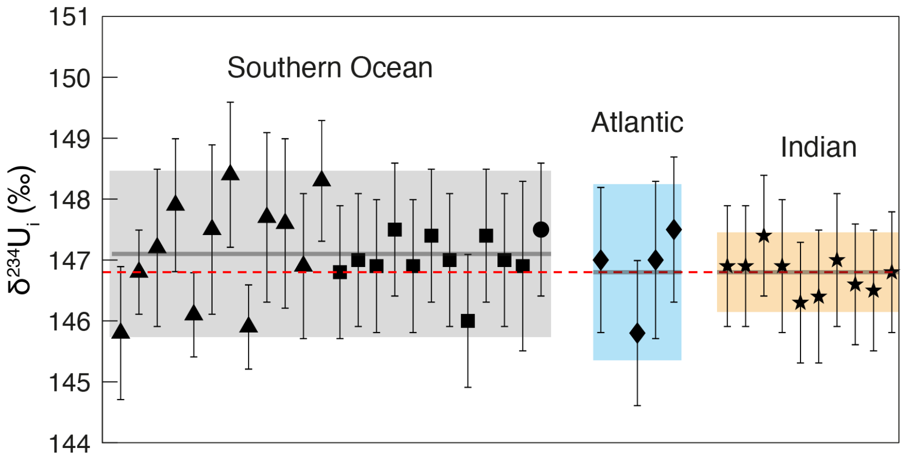

Supplementary Figure 5 | Compiled recent deep-sea coral (<1 ka) $\delta^{234}U_i$ from the Southern Ocean[2-4], Atlantic Ocean[3], and Indian Ocean[6]. Error bars represent 2σ uncertainties. Red dashed line marks the $\delta^{234}U$ value of modern seawater[5].

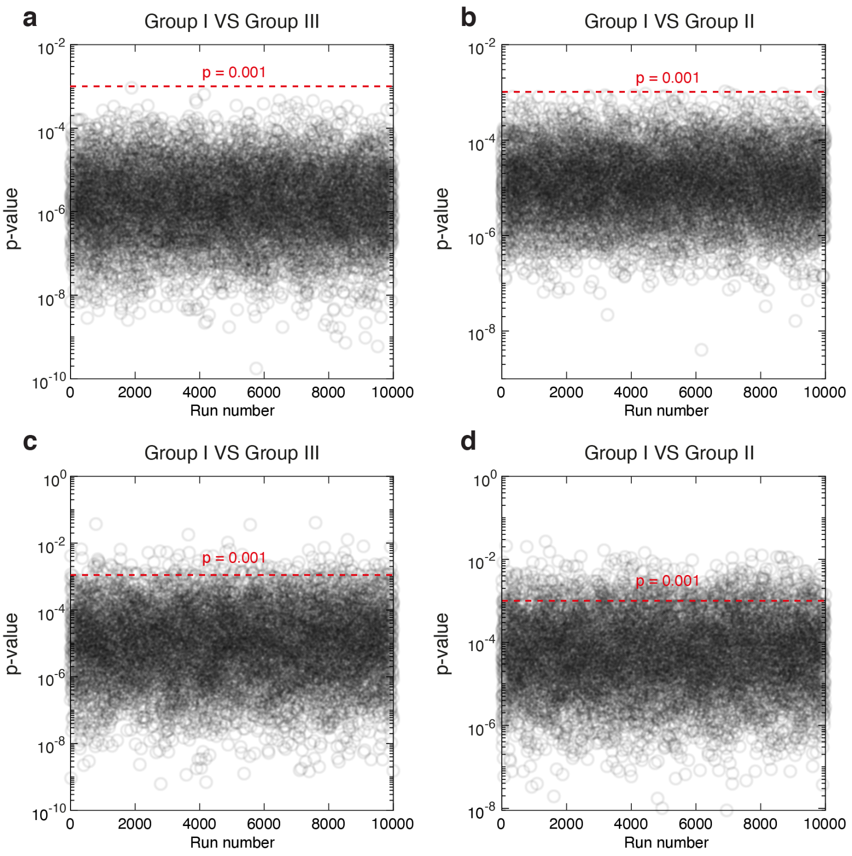

Supplementary Figure 6 | Monte Carlo and Wilcoxon rank sum test. Monte Carlo simulation and Wilcoxon rank sum test were combined to test whether the transient perturbations at 16 – 14 ka are caused by the internal variability of coral $\delta^{234}U$. 10,000 synthetic time series of $\delta^{234}U$ were generated by introducing errors to $\delta^{234}U$ measurements for different groups of corals (Fig. 3b). These errors were represented as normally distributed random numbers with a mean of zero and specified standard deviations (2‰ in **a** and **b**, and 3‰ in **c** and **d**). The *p*-values, which reflect two-sided Wilcoxon rank sum tests, were calculated to compare the $\delta^{234}U$ medians between Group I and Group III (**a** and **c**), as well as between Group I and Group II (**b** and **d**), for each time series. The red dashed lines denote a *p*-value of 10^{-3}.

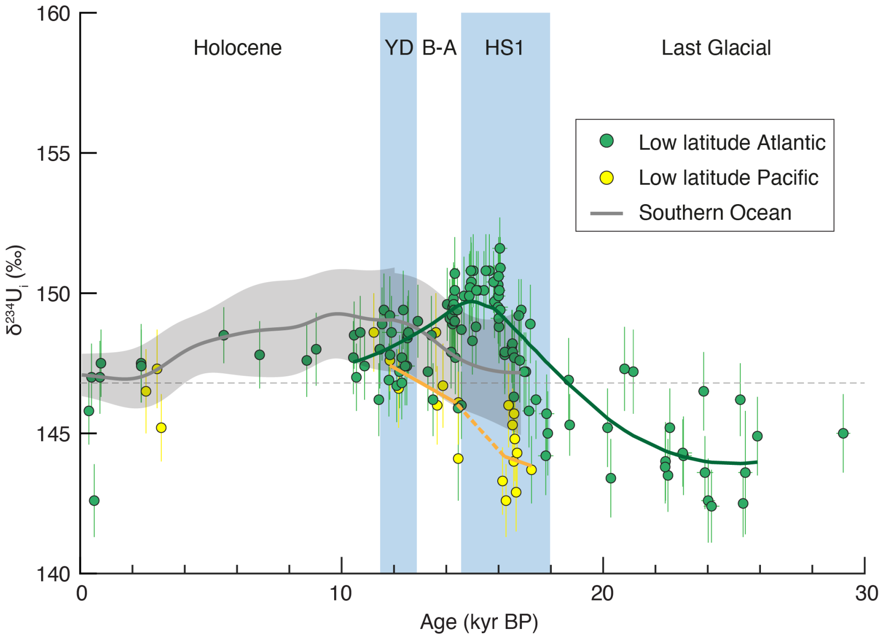

Supplementary Figure 7 | Comparison of Southern Ocean seawater $\delta^{234}U$ record with low latitude Atlantic and Pacific seawater $\delta^{234}U$ records[7]. Only the smoothed Southern Ocean seawater $\delta^{234}U$ record enveloped by grey shadows (± 2σ uncertainties) is shown for better visualization. Green and orange lines represent smoothed low latitude Atlantic and Pacific seawater $\delta^{234}U$ records, respectively. Error bars represent 2σ uncertainties.

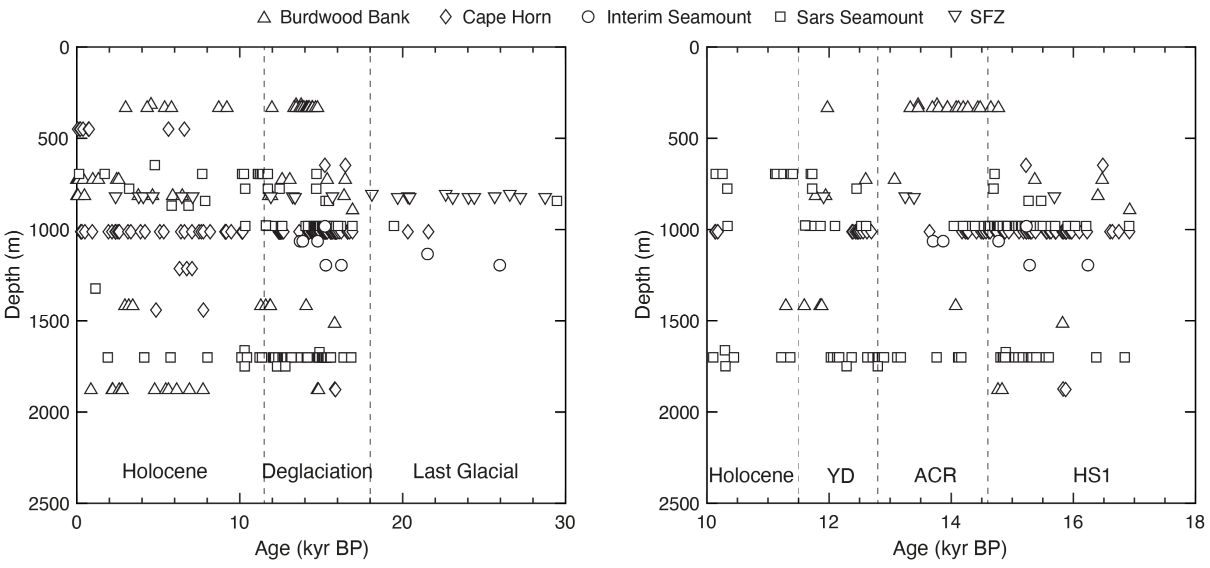

Supplementary Figure 8 | Age-depth distribution for deep-sea corals at Drake Passage. Symbols are the same as in Fig. 1a.

Supplementary References

1. Bradtmiller, L. I., Robinson, L. F., McManus, J. F., Auro, M. E. & Bostock, H. C. The distribution of ^{231}Pa and ^{230}Th in paired water column and surface sediment samples. *Geochimica et Cosmochimica Acta Supplement* **73**, A154 (2009).
2. Burke, A. & Robinson, L. F. The Southern Ocean's Role in Carbon Exchange During the Last Deglaciation. *Science* **335**, 557-561 (2012).
3. Chen, T. *et al.* Synchronous centennial abrupt events in the ocean and atmosphere during the last deglaciation. *Science* **349**, 1537-1541 (2015).
4. Li, T. *et al.* Rapid shifts in circulation and biogeochemistry of the Southern Ocean during deglacial carbon cycle events. *Science Advances* **6**, eabb3807 (2020).
5. Andersen, M., Stirling, C. & Zimmermann, B. Precise determination of the open ocean ^{234}U/^{238}U composition. *Geochemistry Geophysics Geosystems* **11** (2010).
6. Pratt, N. *et al.* Temporal distribution and diversity of cold-water corals in the southwest Indian Ocean over the past 25,000 years. *Deep Sea Research Part I: Oceanographic Research Papers* (2019).
7. Chen, T. *et al.* Ocean mixing and ice-sheet control of seawater ^{234}U/^{238}U during the last deglaciation. *Science* **354**, 626-629 (2016).
