# Supplementary material for: Enhanced subglacial discharge from Antarctica during meltwater pulse 1A
Source: Nat Commun. 2023 Nov 13;14:7327. doi: 10.1038/s41467-023-42974-0 (PMC10643554; doi:10.1038/s41467-023-42974-0)
Supplement: Supplementary file 3 — Description of Additional Supplementary Files [file 41467_2023_42974_MOESM3_ESM.pdf]

Description of Additional Supplementary Files

File Name: Supplementary Data 1
Description: Compiled uranium series data of the deep-sea corals collected from Drake Passage.
